# Establishment of a Rapid Detection System for ISG20-Dependent SARS-CoV-2 Subreplicon RNA Degradation Induced by Interferon-α

**DOI:** 10.3390/ijms222111641

**Published:** 2021-10-28

**Authors:** Yutaka Furutani, Mariko Toguchi, Shoko Higuchi, Kaori Yanaka, Luc Gailhouste, Xian-Yang Qin, Takahiro Masaki, Sae Ochi, Tomokazu Matsuura

**Affiliations:** 1 RIKEN Cluster for Pioneering Research Liver Cancer Prevention Research Unit, Saitama 351-0198, Japan; mariko.takada@riken.jp (M.T.); shiguchi@riken.jp (S.H.); yanakak@riken.jp (K.Y.); luc.gailhouste@riken.jp (L.G.); xyqin@riken.jp (X.-Y.Q.); matsuuratomo@gmail.com (T.M.); 2 Department of Laboratory Medicine, The Jikei University School of Medicine, Tokyo 105-8461, Japan; tmasaki@jikei.ac.jp (T.M.); ochisae1024@gmail.com (S.O.)

**Keywords:** COVID-19, replicon, interferon, interferon-stimulated genes, ISG20, CDM-3008

## Abstract

Inhaled nebulized interferon (IFN)-α and IFN-β have been shown to be effective in the management of coronavirus disease 2019 (COVID-19). We aimed to construct a virus-free rapid detection system for high-throughput screening of IFN-like compounds that induce viral RNA degradation and suppress the replication of severe acute respiratory syndrome coronavirus 2 (SARS-CoV-2). We prepared a SARS-CoV-2 subreplicon RNA expression vector which contained the SARS-CoV-2 5′-UTR, the partial sequence of ORF1a, luciferase, nucleocapsid, ORF10, and 3′-UTR under the control of the cytomegalovirus promoter. The expression vector was transfected into Calu-3 cells and treated with IFN-α and the IFNAR2 agonist CDM-3008 (RO8191) for 3 days. SARS-CoV-2 subreplicon RNA degradation was subsequently evaluated based on luciferase levels. IFN-α and CDM-3008 suppressed SARS-CoV-2 subreplicon RNA in a dose-dependent manner, with IC50 values of 193 IU/mL and 2.54 μM, respectively. HeLa cells stably expressing SARS-CoV-2 subreplicon RNA were prepared and treated with the IFN-α and pan-JAK inhibitor Pyridone 6 or siRNA-targeting ISG20. IFN-α activity was canceled with Pyridone 6. The knockdown of ISG20 partially canceled IFN-α activity. Collectively, we constructed a virus-free rapid detection system to measure SARS-CoV-2 RNA suppression. Our data suggest that the SARS-CoV-2 subreplicon RNA was degraded by IFN-α-induced ISG20 exonuclease activity.

## 1. Introduction

Coronavirus disease (COVID-19) is a worldwide pandemic, and the development of therapeutic agents that specifically target severe acute respiratory syndrome coronavirus 2 (SARS-CoV-2) molecules is long awaited. Although the pandemic has been suppressed by the development of vaccines, it persists, and there are concerns regarding the emergence of mutant strains that weaken vaccine efficacy. In recent years, new coronaviruses, such as Middle East respiratory syndrome coronavirus (MERS-CoV), severe acute respiratory syndrome coronavirus (SARS-CoV), and SARS-CoV-2, have emerged at a frequency of approximately once per decade [1,2]. The development of therapeutic agents that are effective against any coronavirus is important. Therefore, we focused on small chemical compounds that enhance the degradation of SARS-CoV-2 genomic RNA.

Interferons (IFNs) induce interferon-stimulated gene (ISG) expression and exhibit antiviral activities [3]. Thus, we focused on small chemical compounds with IFN-like activity. Type I IFNs such as IFN-α and IFN-β bind to the heterodimeric receptor complex consisting of IFN-α/β receptor 1 (IFNAR1) and IFN-α/β receptor 2 (IFNAR2), thereby resulting in the phosphorylation of Janus kinase 1 (JAK1) and of signal transducers and activators of transcription 1 and 2 (STAT1 and STAT2). After the formation of the STAT1, STAT2, and interferon regulatory factor 9 (IRF9) complex, the transcription of interferon-stimulated genes (ISGs) is induced [3]. CDM-3008 (RO8191) was identified as an orally available anti-hepatitis C virus (HCV) compound from chemical screening using HCV replicon cells [4]. We identified that CDM-3008 suppresses cccDNA in hepatitis B virus (HBV)-infected primary cultured human hepatocytes; thus, we named CDM after the cccDNA modulator [5]. The IFNAR2 agonist CDM-3008 shows anti-HCV, anti-HBV, and anti-Zika virus activity through ISG induction [4,5,6]. Toll-like receptors (TLRs) are pattern-recognition receptors related to viral infections. TLR agonists also induce interferon production, and ISG induction is upregulated in infected cells [7]. Small-molecule compounds with IFN-like activity may be promising therapeutic agents for several viral infections.

It has been reported that IFN signaling is suppressed by the nsp1, nsp6, nsp13, and ORF6 proteins of SARS-CoV-2 [8], and analysis of severe COVID-19 patients has shown that autoantibodies against IFNs are produced, which weakens their effect [9,10]. Additionally, mutations in IRF9, IFNAR1, and IFNAR2 were observed in severe patients, which implies weakened IFN signaling and may have contributed to disease severity due to the failure to suppress SARS-CoV-2 replication [11]. A genome-wide association study (GWAS) of severe COVID-19 patients showed a significant association between disease severity and decreased IFNAR2 expression levels [12]. The therapeutic efficacy of IFN-α and IFN-β has been debated, and it has been reported that IFN-β1a has no therapeutic effect when administered subcutaneously or intravenously [13]. Meanwhile, inhaled nebulized IFN-α2b or IFN-β1a has a therapeutic effect and accelerates recovery from COVID-19 [14,15,16]. A phase II clinical trial using inhaled nebulized IFN-β1a (SNG001) for the treatment of COVID-19 showed that the SNG001-treated group of patients recovered more rapidly than the placebo group [14]. Moreover, a phase III clinical trial of SNG001 (NCT04732949) is ongoing, and it will confirm whether SNG001 can accelerate the recovery of hospitalized patients receiving oxygen. Therefore, IFN-α and IFN-β are useful for the treatment of COVID-19, but they cannot exert their anti-SARS-CoV-2 activity without supplying sufficient amounts to respiratory epithelial cells to induce ISG expression.

Moreover, to perform experiments using SARS-CoV-2, we need to use a biosafety level 3 facility (BSL-3), which is not suitable for the large-scale screening of small chemical compounds. The replicon system has been shown to be a powerful tool for screening small chemical compounds [17,18]. SARS-CoV and SARS-CoV-2 replicons have been constructed, and anti-SARS-CoV-2 drugs have been confirmed by activity [19,20,21,22].

Therefore, we developed a virus-free method to measure the degradation of SARS-CoV-2 RNA by IFN-α within a short interval. Using this method, we were able to screen new compounds that induce the degradation of SARS-CoV-2 genomic RNA.

## 2. Results

**Figure 1:**
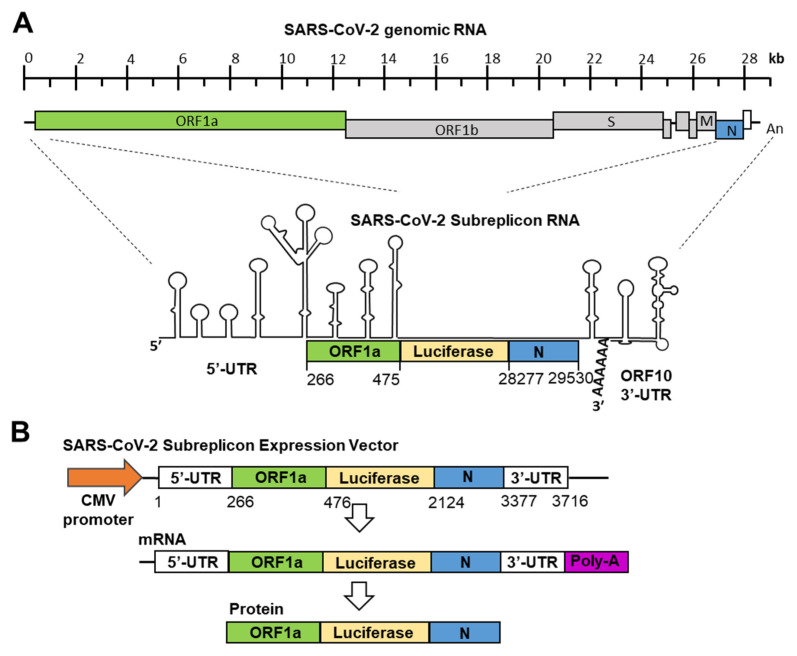
Construction of SARS-CoV-2 subreplicon RNA expression vector. (**A**) Schematic structure of SARS-CoV-2 genomic RNA and SARS-CoV-2 subreplicon RNA. SARS-CoV-2 subreplicon RNA expression vector contains 5′-UTR (1–265), a part of ORF1a (266–475, green box), nucleocapsid (N, 28277–29530, blue box), and ORF10 (open box) and 3′-UTR (29531–29870), and the firefly luciferase sequence was inserted between ORF1a and nucleocapsid. The secondary structure of SARS-CoV-2 genomic RNA presented by Wacker et al. is schematically shown [23], and the protein translated regions of ORF1a, luciferase, and nucleocapsid are boxed and colored with green, yellow, and blue, respectively. Spike (S), membrane (M), and nucleocapsid (N) regions are boxed and marked S, M, and N, respectively. SARS-CoV-2 subreplicon RNA is numbered based on the Gene Bank MN994467 SARS-CoV-2 genomic RNA sequence. (**B**) SARS-CoV-2 subreplicon cDNA was inserted into the CMV promoter (orange arrow) for construction of the expression vector. Lengths of SARS-CoV-2 subreplicon cDNA regions are numbered. SARS-CoV-2 subreplicon RNA is expressed with poly-A tail (purple box). A fusion protein of part of ORF1a, firefly luciferase, and nucleocapsid is translated from the mRNA.

SARS-CoV-2 genomic RNA forms a stem-loop containing double-stranded RNA regions at the 5′-end and 3′-end, similar to SARS-CoV and MARS-CoV (Figure 1A) [23,24]. IFN-α binds to heterodimers of IFNAR1 and IFNAR2 and induces the expression of ISGs. Among the ISGs, OAS1, 2, and 3 and ISG20 are thought to modify SARS-CoV-2 genomic RNA and induce its degradation [25]. In this study, we constructed a system for measuring the SARS-CoV-2 genomic RNA degradation activity of IFN-α within 1 day.

The production of SARS-CoV and SARS-CoV-2 replicons has also been reported [20,26]. Since the RNA genome of SARS-CoV-2 is approximately 30 kb, it is difficult to prepare a replicon that stably expresses the full-length viral RNA in cultured cells. Therefore, it is considered unsuitable as a screening system. To specialize in SARS-CoV-2 genomic RNA degradation induced by IFN-α, we focused on virus-specific stem-loops containing double-stranded RNA structures at the 5′- and 3′-ends (Figure 1A). The minimal sequence required for coronaviruses’ genomic RNA replication has been shown in previous studies [27], and based on coronaviruses’ minimal sequence, we constructed a SARS-CoV-2 subreplicon RNA expression vector. SARS-CoV-2 subreplicon RNA contains 5′-UTR, a part of ORF1a, the luc2 gene, nucleocapsid, ORF10, and 3′-UTR and is expressed under the control of the CMV promoter. The total length obtained was 3.7 kb, and it was possible to insert it into an expression vector under the control of a CMV promoter. In addition, the expression vector was ≤10 kb, and stable cell lines were easily produced. After the transcription of SARS-CoV-2 subreplicon RNA, the fusion protein containing a part of ORF1, luciferase, and nucleocapsid was transcribed (Figure 1B). The expression levels of SARS-CoV-2 subreplicon RNA were measured using a luciferase assay. In addition, SARS-CoV-2 subreplicon RNA replication could be achieved by adding RNA-dependent RNA polymerase to this sequence.

**Figure 2:**
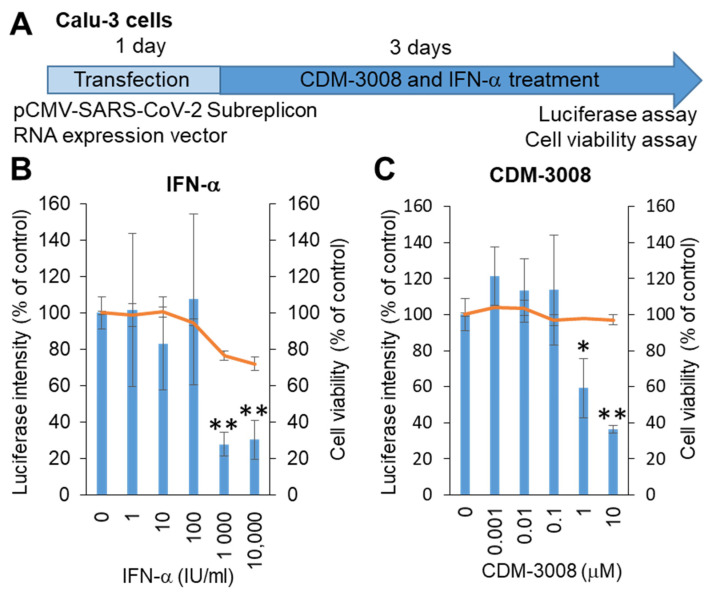
Measurement of IFN-α and CDM-3008 anti-SARS-CoV-2 activity using SARS-CoV-2 subreplicon RNA-expressing Calu-3 cells. (**A**) Schematic of the experimental design of transfection with SARS-CoV-2 subreplicon RNA expression vector and treatments of IFN-α and CDM-3008 for 3 days prior to luciferase and cell viability assays. (**B**,**C**) SARS-CoV-2 subreplicon RNA-expressing Calu-3 cells were treated with 1–10,000 IU/mL IFN-α in B and 0.001-10 μM CDM-3008 in C for 1 day. Luciferase intensity (blue bars) and cell viability (orange lines) were measured and are shown as % of DMSO control. Error bars indicate SD (*n* = 3). * *p* < 0.05 and ** *p* < 0.01 (two-tailed *t*-test).

SARS-CoV-2 mainly infects the respiratory organs. Hoffmann et al. showed that pseudotyped viruses for SARS-CoV-2 efficiently enter Calu-3, Caco-2, and Vero cells [28]. Thus, we selected Calu-3 cells derived from lung adenocarcinoma, and the SARS-CoV-2 subreplicon RNA expression vector was transfected into Calu-3 cells. After 1 day of transfection, the cells were treated with IFN-α or the IFNAR2 agonist CDM-3008 for 3 days, and cell viability and luminescence intensity were measured (Figure 2A). As a result, the IC_50_ of IFN-α was 193 IU/mL, and the CC_50_ was more than 10 μM (Figure 2B), while the IC_50_ of CDM-3008 was 2.54 μM, without showing cytotoxicity (Figure 2C). Therefore, similar to IFN-α, CDM-3008 showed anti-SARS-CoV-2 activity through the degradation of SARS-CoV-2 genomic RNA.

**Figure 3:**
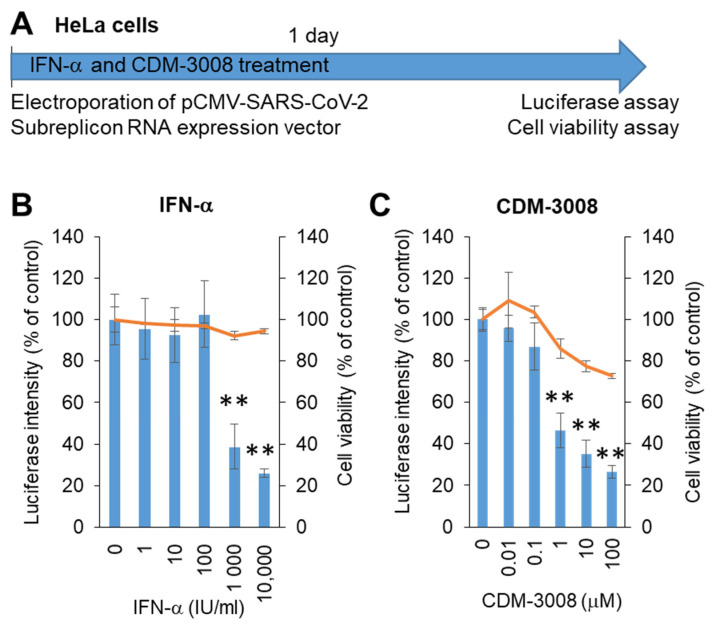
Measurement of IFN-α and CDM-3008 anti-SARS-CoV-2 activity using SARS-CoV-2 subreplicon expression vector-electroporated HeLa cells. (**A**) Schematic of the experimental design of electroporation with SARS-CoV-2 subreplicon expression vector and treatments of IFN-α and CDM-3008 for 1 day prior to luciferase and cell viability assays. (**B**,**C**) SARS-CoV-2 subreplicon-expressing HeLa cells were treated with 1–10,000 IU/mL IFN-α in B and 0.01-100 μM CDM-3008 in C for 1 day. Luciferase intensity (blue bars) and cell viability (orange lines) were measured and are shown as % of DMSO control. Error bars indicate SD (*n* = 3). * *p* < 0.05 and ** *p* < 0.01 (two-tailed *t*-test).

The growth rate of Calu-3 cells was not higher than that of HeLa cells, and Calu-3 cells were not used for electroporation. Thus, we changed the Calu-3 cells to HeLa cells to construct a screening system. The SARS-CoV-2 subreplicon RNA expression vector was electroporated into HeLa cells and simultaneously treated with IFN-α or the IFNAR2 agonist CDM-3008 for 1 day, and cell viability and luminescence intensity were measured (Figure 3A). The IC_50_ values of IFN-α and CDM-3008 were 667 IU/mL, without showing cytotoxicity (Figure 3B), and the IC_50_ and CC_50_ of CDM-3008 were 0.78 μM and >100 μM, respectively (Figure 3C). Therefore, the activity of IFN-α and CDM-3008 in HeLa cells was comparable to that in Calu-3 cells, and the assay was performed within 1 day. However, CDM-3008 reduced cell viability to 72.8% at 100 μM, while IFN-α did not. Thus, we selected IFN-α as a positive control for chemical screening of HeLa cells.

**Figure 4:**
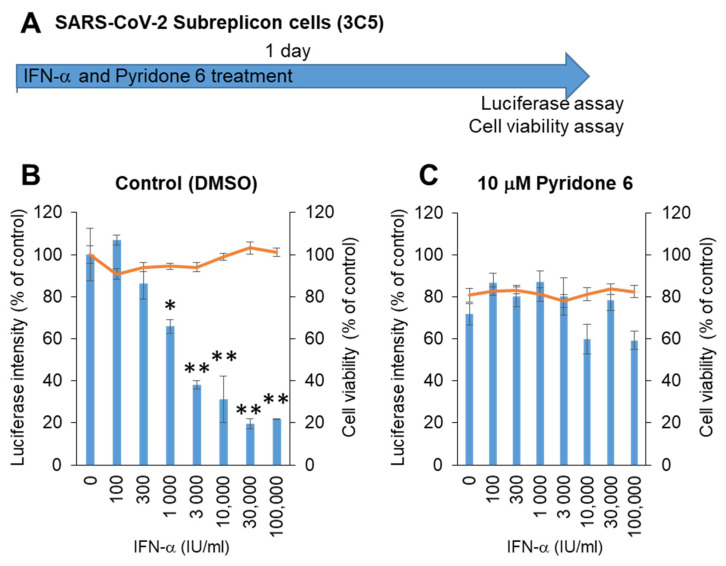
Measurement of IFN-α anti-SARS-CoV-2 activity using HeLa cells stably expressing SARS-CoV-2 subreplicon. (**A**) Schematic of the experimental design of treatments of HeLa cells stably expressing SARS-CoV-2 subreplicon (3C5) with IFN-α and Pyridone 6 for 1 day prior to luciferase and cell viability assays. (**B**,**C**) HeLa cells stably expressing SARS-CoV-2 subreplicon were treated with 100–100,000 IU/mL IFN-α in B and simultaneously treated with 10 μM Pyridone 6 in C for 1 day. Luciferase intensity (blue bars) and cell viability (orange lines) were measured and are shown as % of DMSO control. Error bars indicate SD (*n* = 3). * *p* < 0.05 and ** *p* < 0.01 (two-tailed *t*-test).

Next, stable SARS-CoV-2 subreplicon RNA-expressing HeLa cells were prepared using an expression vector containing a neomycin-resistant gene. After selection with 1 mg/mL G418, the cells were plated and simultaneously treated with IFN-α for 1 day (Figure 4A). The luminescence intensity was suppressed by IFN-α in a dose-dependent manner, with an IC_50_ of 1875 IU/mL without cytotoxicity (Figure 4B). IFN-α activity was suppressed by the pan-JAK inhibitor Pyridone 6 (Figure 4C), suggesting that IFN-α induced the expression of ISGs by activating JAK/STAT, and ISGs enhanced the degradation of SARS-CoV-2 subreplicon RNA. The cells treated with 10 μM Pyridone 6 had approximately 80% cell viability, suggesting that 10 μM Pyridone 6 had weak cytotoxicity in HeLa cells.

**Figure 5:**
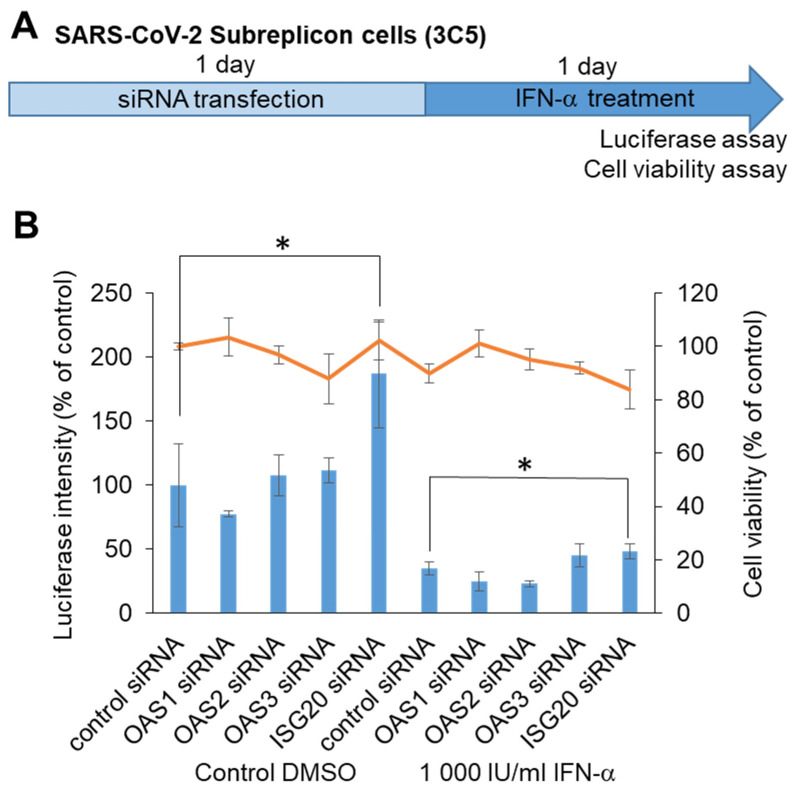
Determination of ISGs suppressing SARS-CoV-2 genomic RNA using siRNA. (**A**) Schematic of the experimental design of HeLa cells stably expressing SARS-CoV-2 subreplicon (3C5) treated with siRNA and IFN-α for 1 day each prior to luciferase and cell viability assays. (**B**) HeLa cells stably expressing SARS-CoV-2 subreplicon were treated with OAS1, 2, and 3 and ISG20 siRNA for 1 day, and then the cells were treated with 1000 IU/mL IFN-α for 1 day. Luciferase intensity (blue bars) and cell viability (orange line) were measured and are shown as % of control siRNA-treated DMSO control. Error bars indicate SD (*n* = 3). * *p* < 0.05 (two-tailed *t*-test).

To identify the target molecule for chemical screening, we performed knockdown of RNA-editing enzymes within ISGs, which was able to induce SARS-CoV-2 subreplicon RNA degradation. We assumed that ISGs such as OAS1, 2, and 3 and ISG20, which detect virus-derived RNA and promote degradation, enhance the degradation of SARS-CoV-2 subreplicon RNA. We knocked down OAS1, 2, and 3 and ISG20 using siRNAs in a SARS-CoV-2 subreplicon RNA-expressing stable cell line, and then the cells were treated with IFN-α for 1 d (Figure 5A). Treatment with ISG20 siRNA, even without IFN-α, increased the luminescence intensity. ISGs were thought to be induced by the IFN-α-like substance contained in the cell culture medium, and SARS-CoV-2 subreplicon RNA was constantly degraded. The degradation was suppressed by ISG20 siRNA (Figure 5B). Furthermore, the luminescence intensity was suppressed by 1000 IU/mL IFN-α, and this effect was partly canceled by ISG20 siRNA. These results suggest that SARS-CoV-2 genomic RNA is degraded by ISG20.

## 3. Discussion

In this study, we developed an experimental system that easily shows the anti-SARS-CoV-2 activity of IFN-α within 1 day. Using this experimental system, we were able to identify small-molecule compounds that promote the degradation of SARS-CoV-2 genomic RNA. We confirmed the inhibitory effect of the IFNAR2 agonist CDM-3008, a small chemical compound, in Calu-3 and HeLa cells. This experimental system is optimal for screening compounds that promote the degradation of SARS-CoV-2 genomic RNA.

### 3.1. Therapeutic Effect of IFNs against SARS-CoV-2 In Vivo and In Vitro

To analyze the pharmacological effects of IFNs in vivo, several coronavirus-infected animal models were used. Using MERS-CoV-infected mice expressing hDPP4, IFN-β and IFN-γ suppressed MERS-CoV [29]. In the MERS-CoV-infected common marmoset model, MERS-CoV levels were lower in the IFN-β1b-treated group than those in untreated animals [30]. In MERS-CoV-infected rhesus macaques, a combinational treatment with ribavirin and IFN-α2b reduced MERS-CoV levels as compared to untreated animals [31]. Using ACE2-transgenic mice, IFN-λ1a showed an inhibitory effect on SARS-CoV-2 [32]. These in vivo analyses of animal models support the use of IFNs in the treatment of MERS and COVID-19.

In the activity measurement using the SARS-CoV-2 subreplicon RNA in Calu-3 cells, the IC_50_ value of IFN-α was 193 IU/mL. The IC_50_ of IFN-α in SARS-CoV-2-infected Calu-3 cells was less than 100 U/mL [33]. This may have occurred because the activity measurement using SARS-CoV-2-infected Calu-3 cells was performed after 16 h of pretreatment with IFN-α before infection, whereas IFN-α treatment of SARS-CoV-2 subreplicon RNA was performed 24 h after transfection. Collectively, the in vitro analysis of IFN-α using Calu-3 cells also supports the use of IFN-α for the treatment of COVID-19.

### 3.2. SARS-CoV-2 Replicon System

Replicons have been produced for high-throughput screening of SARS-CoV and SARS-CoV-2. Hertzig et al. constructed SARS-CoV replicon RNA consisting of 5′-UTR, ORF1a, ORF1b, GFP or luciferase, nucleocapsid, and 3′-UTR and used this system for IFN-α activity assays (IC_50_ = 10 U/mL) [21]. Similar replicon RNA was constructed using the SARS-CoV-2 genomic RNA sequence and was used for high-throughput screening [20,26,34,35]. In addition, Luo et al. constructed three expression vectors containing SARS-CoV-2 genes nsp1-16 and expressed these genes with SARS-CoV-2 replicon RNA consisting of 5′-UTR, IRES, GFP, transcription regulatory sequence, luciferase, and 3′-UTR [22]. Vial et al. showed that the SARS-CoV-2 proteins required for the synthesis of positive strands from negative-strand replicon RNA are Nsp7, 8, and 12. Using these systems, they confirmed the anti-SARS-CoV-2 activity of remdesivir [36]. From these studies, it was concluded that the 5′-UTR, 3′-UTR, and reporter genes such as GFP and luciferase are required to produce SARS-CoV-2 replicon RNA, and that RNA-dependent RNA polymerases (RdRp, nsp12) are required for replication. In contrast, in our experimental system for measuring the activity of IFNs, only the positive strand of SARS-CoV-2 subreplicon RNA is necessary because we are measuring the degradation of RNA, and the action of RdRp is unnecessary. Thus, our SARS-CoV-2 subreplicon system is simpler and more compact than the previously reported SARS-CoV-2 replicon systems.

### 3.3. SARS-CoV-2 Subreplicon RNA Degradation Mechanism

In this study, we showed that ISG20 is involved in the degradation of the SARS-CoV-2 RNA genome (Figure 5). ISG20 is a 20 kDa exonuclease that degrades single-stranded RNA and DNA [37]. Imam et al. found that the epsilon stem-loop of N6-methyladenosine (m^6^A)-modified HBV pregenomic RNA (pgRNA) is recognized by the m6A reader protein YTH-domain family 2 (YTHDF2), and that degradation is induced by a complex of YTHDF2 and ISG20 [38]. Additionally, Liu et al. assessed the SARS-CoV-2 m^6^A methylome and showed that m^6^A sites are mainly accumulated in the regions of nucleocapsid, ORF10, and 3′-UTR in positive-sense genomic RNA. M^6^A methylation is increased in negative-sense genomic RNA in the 5′-UTR and ORF1a regions from 24 to 56 h after infection [39]. SARS-CoV-2 subreplicon RNA is a positive strand because transcription by RNA-dependent polymerases does not occur in our system. Thus, it is considered that the nucleocapsid, ORF10, and 3′-UTR regions of SARS-CoV-2 subreplicon RNA are modified by m^6^A, recognized by YTHDF2, and degraded by ISG20. In addition, during the IFN-β response, many mRNAs encoding ISGs are modified by m^6^A. YTHDF1 binds to m^6^A-modified mRNA and enhances transcription [40]. This suggests that type I IFNs and CDM-3008 induce ISG20 mRNA expression and simultaneously promote translation of ISG20, and that degradation of SARS-CoV-2 genomic RNA could be enhanced by ISG20 and YTHDF1 complexes. The development of a SARS-CoV-2 treatment method that promotes m^6^A modification and ISG20 activation induced by type I IFNs and CDM-3008 should be further explored.

## 4. Materials and Methods

### 4.1. Construction of pCMV-SARS-CoV-2 Subreplicon RNA Expression Vector

To construct the SARS-CoV-2 subreplicon RNA expression vector, 5′-UTR (1–265), a part of ORF1a (266–475), nucleocapsid (N, 28277–29530), and ORF10 and 3′-UTR (29531–29870) were selected from the SARS-CoV-2 genome sequence (GenBank: MN994467), and the firefly luciferase sequence (luc2 gene, Promega, Madison, WI, USA) was inserted between ORF1a and the nucleocapsid.

DNA fragments containing 5′-UTR, ORF1a, nucleocapsid, ORF10, and 3′-UTR of the SARS-CoV-2 genome sequence and the luc2 gene (Promega) were synthesized in vitro using an artificial gene synthesis service (FASMAC, Atsugi, Japan) (Figure 1). The fragment was amplified using 5′-TCACATGGCTCGACAGATCTATTAAAGGTTTATACCTTCCC-3′ and 5′-CGGATCGATCCTTATCGGATGTCATTCTCCTAAGAAGCTATT-3′. A partial sequence of the pIRES vector (Takara Bio, Kusatsu, Japan) was amplified using 5′-ATCCGATAAGGATCGATCCG-3′ and 5′-AGATCTGTCGAGCCATGTGA-3′. The SARS-CoV-2 subreplicon sequence was fused between the CMV promoter and the f1 ori using the In-Fusion Snap Assembly Master Mix (Takara Bio).

### 4.2. Cell Culture

Calu-3 cells were maintained in MEM (M4526-500ML, Sigma-Aldrich, St. Louis, MO, USA) supplemented with 10% fetal bovine serum, 2 mM L-glutamine (25030-149, Thermo Fisher, Waltham, MA, USA), 100 U/mL penicillin, and 100 μg/mL streptomycin (26253-84, Nacali Tesque, Kyoto, Japan). HeLa cells were maintained in DMEM high glucose (08459-64, Nacali Tesque) supplemented with 10% fetal bovine serum, 100 U/mL penicillin, and 100 μg/mL streptomycin (26253-84, Nacali Tesque) in 5% CO_2_ at 37 °C.

### 4.3. Lipofection

Calu-3 cells (100 μL of 1.5 × 10^5^ cells/mL) were mixed with 100 ng of the pCMV-SARS-CoV-2 subreplicon RNA expression vector and 0.3 μL of X-tremeGene 360 transfection reagent (8724121001, Roche, Basel, Switzerland) and plated on a 96-well white cell culture plate (655083, Greiner Bio-One, Kremsmunster, Austria) and a 96-well clear cell culture plate (92096, TPP, Trasadingen, Switzerland). After 1 day of transfection, the cells were treated with 1–10,000 IU/mL IFN-α and 0.001–10 µM CDM-3008 for 3 days.

### 4.4. Electroporation

HeLa cells (400 μL of 2 × 10^6^ cells/mL) were mixed with 40 μg of the pCMV-SARS-CoV-2 subreplicon expression vector, which was subsequently electroporated at 250 V, 500 μF, and ∞ Ω, in a 4 mm-gap cuvette, using Gene Pulser Xcell equipped with a CM module (Bio-Rad, Hercules, CA, USA). The cells (1 × 10^5^ cells/well) were plated on a 96-well white cell culture plate (655083, Greiner Bio-One) and 96-well clear cell culture plates (92096, TPP).

### 4.5. Stable Cell Line for SARS-CoV2 Subreplicon RNA

The pCMV-SARS-CoV-2 subreplicon RNA expression vector was digested with BamHI overnight. After purification of the vector using Qiaquick PCR purification (Qiagen, Venlo, The Netherlands), 2 µg of the digested vector and 6 µL of X-tremeGene 360 transfection reagent (Roche) were mixed and added to HeLa cells cultured on a 35 mm plate. After 3 days, the HeLa cells were replated and selected with 1 mg/mL G418-containing medium. After 2 weeks of selection, the SARS-CoV2 subreplicon 3C5 cell line was selected.

### 4.6. Luciferase Assay

After treatment, the luciferase intensity was measured using the Steady-Glo Luciferase Assay System (Promega), according to the manufacturer’s instructions. Briefly, the cells were mixed with 50 μL of cell culture medium and Steady Glo reagent (Promega) for 10 min at 25 °C using a plate shaker. Luciferase intensity was measured using a multimode plate reader (EnSight, PerkinElmer, Waltham, MA, USA).

### 4.7. Cell Viability Assay (XTT Assay)

After treatment, cell viability was assayed using the Cell Proliferation Kit II (Roche) according to the manufacturer’s instructions. Briefly, 100 μL of cell culture medium and 50 μL of XTT reagent (Cell Proliferation Kit II, Roche) under 5% CO_2_ at 37 °C for 45 min were added to the cells. The absorbance at 492 nm was measured using a multimode plate reader (EnSight, PerkinElmer).

### 4.8. Transfection of siRNA

SARS-CoV2 subreplicon 3C5 cells (2 × 10^4^ cells/well) were mixed with OAS1, OAS2, OAS3, and ISG20 siRNA (ON-TARGET SMARTpool siRNA, Horizon Discovery, Cambridge, UK), and Lipofectamine RNAi Max transfection reagent (Thermo Fisher). The cells were then plated on a 96-well white cell culture plate (655083, Greiner Bio-One) and a 96-well clear cell culture plate (92096, TPP). After 1 day of transfection, the cells were treated with 1000 IU/mL IFN-α for 1 day.

### 4.9. Statistical Analysis

Statistical analysis was performed using Microsoft Office 2016 (Microsoft, Redmond, WA, USA).

## 5. Conclusions

The advantages of small-molecule compounds with IFN-like activity that are available in oral and inhalation formulations include innate immune response activation in the host and the inhibition of various viruses. To measure anti-SARS-CoV-2 activity through RNA genome degradation, we constructed a SARS-CoV-2 subreplicon RNA expression vector. IFN-α and CDM-3008 showed anti-SARS-CoV-2 activity in SARS-CoV-2 subreplicon RNA-expressing Calu-3 and HeLa cells. We constructed a virus-free screening system to select small chemical compounds with IFN-like activity. It is important to screen for new compounds using this system and to develop several therapeutic drugs for future pandemics.

## Data Availability

The data presented in this study are available in the article.
